# Under-vaccinated groups in Europe and their beliefs, attitudes and reasons for non-vaccination; two systematic reviews

**DOI:** 10.1186/s12889-018-5103-8

**Published:** 2018-01-30

**Authors:** N. Fournet, L. Mollema, W. L. Ruijs, I. A. Harmsen, F. Keck, J. Y. Durand, M. P. Cunha, M. Wamsiedel, R. Reis, J. French, E. G. Smit, A. Kitching, J. E. van Steenbergen

**Affiliations:** 1 Centre for Infectious Diseases Control, National Institute for Public Health and the Environment (RIVM), Bilthoven, The Netherlands; 2 Municipal Health Service (GGD) Amsterdam, Amsterdam, The Netherlands; 3 Laboratoire d’anthropologie sociale - Centre National de la Recherche Scientifique, Paris, France; 4 Centre for Research in Anthropology, Universidade do Minho (CRIA - UMinho), Braga, Portugal; 5 Department of Public Health at Xi’an Jiaotong-Liverpool University, Suzhou, China; 6 Leiden University Medical Centre, Leiden, The Netherlands; 7 Amsterdam Institute for Social Science Research, University of Amsterdam, Amsterdam, The Netherlands; 8 The Children’s Institute, University of Cape Town, Cape Town, South Africa; 9 Strategic Social Marketing, Liphook, UK; 10 Brighton University Business School, Brighton, UK; 11 Amsterdam School of Communication Research, University of Amsterdam, Amsterdam, The Netherlands; 12 Department of Public Health, Health Service Executive, St Finbarr’s Hospital, Cork, Republic of Ireland; 13 Centre for Infectious Diseases, Leiden University Medical Centre, Leiden, The Netherlands; 14 National Institute for Public Health and the Environment, Epidemiology and Surveillance Unit, P.O. Box 1 (internal P.O. Box 75), 3720 BA Bilthoven, the Netherlands

**Keywords:** Under-vaccinated groups, Low vaccination coverage, Vaccine preventable diseases, Attitude regarding vaccination, Beliefs vaccine, Europe, Religion, Anthroposophic, Roma, Irish Travellers

## Abstract

**Background:**

Despite effective national immunisation programmes in Europe, some groups remain incompletely or un-vaccinated (‘under-vaccinated’), with underserved minorities and certain religious/ideological groups repeatedly being involved in outbreaks of vaccine preventable diseases (VPD).

Gaining insight into factors regarding acceptance of vaccination of ‘under-vaccinated groups’ (UVGs) might give opportunities to communicate with them in a trusty and reliable manner that respects their belief system and that, maybe, increase vaccination uptake. We aimed to identify and describe UVGs in Europe and to describe beliefs, attitudes and reasons for non-vaccination in the identified UVGs.

**Methods:**

We defined a UVG as a group of persons who share the same beliefs and/or live in socially close-knit communities in Europe and who have/had historically low vaccination coverage and/or experienced outbreaks of VPDs since 1950. We searched MEDLINE, EMBASE and PsycINFO databases using specific search term combinations. For the first systematic review, studies that described a group in Europe with an outbreak or low vaccination coverage for a VPD were selected and for the second systematic review, studies that described possible factors that are associated with non-vaccination in these groups were selected.

**Results:**

We selected 48 articles out of 606 and 13 articles out of 406 from the first and second search, respectively. Five UVGs were identified in the literature: Orthodox Protestant communities, Anthroposophists, Roma, Irish Travellers, and Orthodox Jewish communities. The main reported factors regarding vaccination were perceived non-severity of traditional “childhood” diseases, fear of vaccine side-effects, and need for more information about for example risk of vaccination.

**Conclusions:**

Within each UVG identified, there are a variety of health beliefs and objections to vaccination. In addition, similar factors are shared by several of these groups. Communication strategies regarding these similar factors such as educating people about the risks associated with being vaccinated versus not being vaccinated, addressing their concerns, and countering vaccination myths present among members of a specific UVG through a trusted source, can establish a reliable relationship with these groups and increase their vaccination uptake. Furthermore, other interventions such as improving access to health care could certainly increase vaccination uptake in Roma and Irish travellers.

## Background

Vaccination programmes have been shown to reduce health inequality worldwide [1]. However, despite national immunisation programmes in Europe, some groups remain incompletely or un-vaccinated (“under-vaccinated”), with underserved minorities and certain religious/ideological groups repeatedly being involved in outbreaks of vaccine preventable diseases (VPD) [2].

As an example, in 2004, a rubella outbreak occurred within an under-vaccinated religious community in the Netherlands [3], which spread to Canada [4, 5] and led to cases of congenital rubella syndrome [4, 6]. These outbreaks in under-vaccinated groups sometimes cause “spill over” disease in the general population as occurred during two measles outbreaks. One occurred in Germany in 2008, from the anthroposophic community to the general population who had vaccination coverage below the World Health Organisation (WHO) recommended level [7]. The second one, in the Netherlands in 1999–2000, started among unvaccinated members of Orthodox Protestant Reformed churches and spread to children of vaccinating parents, but whose children were susceptible as they were still too young to be vaccinated [8, 9]. Between May 2013 and February 2014, another measles outbreak was ongoing in the Netherlands among the same religious community with 2700 reported cases [10].

The World Health Organization Regional Office for Europe (WHO/EURO) has set several goals for elimination of endemic measles and rubella in Europe [11]. However, achieving this goal and improving VPD vaccination coverage in general remains difficult as long as clusters of large under-vaccinated groups (UVG) still exist in various countries. In addition, in case of a major vaccine preventable outbreak, like the 2009 influenza A(H1N1) pandemic, these groups are likely to refuse any new vaccination which may be advised by the government/public health authorities. Therefore, they can form a susceptible pool of individuals at increased risk to acquire the VPD, and can act as a focus for and multiplier of the infectious agent, with subsequent spread to the general population.

For communicable disease control it is especially the social clustering of non-vaccinated individuals that increases outbreak risk. Clustering of non-vaccinated individuals is found in various groups in Europe. Most of these UVGs are labelled as ‘hard-to-reach’. However, not all groups are hard-to-reach and each group has its specific reasons and even individuals with in a group may differ for which specific approaches are needed and not general ones. Knowledge on the specific reasons for low vaccination uptake among the various UVGs, might facilitate communication based on their (information) needs. The Strategic Advisory Group of Experts (SAGE) on immunization has defined determinants of vaccine hesitancy (i.e. this term refers to delay in acceptance or refusal of vaccines despite availability of vaccination services) worldwide, both for general populations and/or groups. They divided the determinants into three categories: 1. Contextual influences (e.g. religion/culture/gender/socio-economic, communication and media environment), 2. Individual and group influences (e.g. personal experience with vaccination, health system and providers trust), and 3. Vaccine/vaccination specific issues (e.g. risk/benefit (epidemiologic and scientific evidence), costs) [12]. In this study we focus on the specific determinants of vaccination uptake in under-vaccinated groups, which can also include determinants of poor availability. While the only intervention to increase vaccination uptake addressed in this study is on communication, we do recognize that communication might not be the only intervention needed, and that other interventions such as changes in the delivery strategy of vaccines might have high impact. But nevertheless, in all cases communication is essential.

This study is part of one of the Work Packages (WP) of the EU-funded project “Effective Communication in Outbreak Management: development of an evidence-based tool for Europe”, which started in 2011. The aim of our contribution to the project is to identify vantage points for communication strategies and present suggestions for communication with UVGs that can be used effectively by health professionals and agencies throughout Europe, in case of an epidemic or pandemic of a VPD. To that purpose the focus in this paper is on how to identify the UVGs in Europe and the description of factors (beliefs, attitudes and reasons) for poor uptake of vaccination in order to know with whom and how to communicate.

We performed two systematic reviews as to our knowledge this has not been done before. The objective of the first review was how to identify UVGs in Europe and to describe the UVGs, and of the second to describe beliefs, attitudes and reasons of non-vaccination of these UVGs identified by the first review and a comparison of the factors among the UVGs (both qualitative and quantitative studies were included).

## Methods

### Protocol and registration

No review protocol exists and the review has not been registered.

### Eligibility criteria

For the first systematic review to identify UVGs we used the following case definition:

We defined a UVG as a group of persons:

1. Who share the same beliefs and/or who live in socially close-knit communities in Europe
  AND
2. Who have/had historically low vaccination coverage (i.e. below the threshold level needed to eradicate a certain disease) and/or experienced outbreaks of VPD since 1950.

Note, the general population might also have had historically low vaccination uptake such as was the case for MMR in the UK due to a suggested link between autism and the MMR vaccine. However, in this study the focus is on UVGs and not on the general population. Furthermore, we did not include people living together in closed settings as prisons or nursing homes because, although there might be low vaccination coverage reported in some of these settings, they are not identified as a group which is culturally close and/or do not share the same belief system. In addition, there also are some groups who are (partly) refusers or hard-to-reach but are not easily identifiable. For example, the ‘middle-class worried who read something on the internet’ may be only a loosely definable group, but they may still be significant. We also did not include these groups.

In order to find out how to identify UVGs in the literature terms were used that describe a group that shares the same beliefs and/or who lives in socially close-knit communities. Furthermore, we restricted the search to terms such as vaccination and immunisation and did not search for health in general of which vaccination may be part of. Both qualitative and quantitative studies were included. English published articles were selected between 1950 (when many national European immunisation programmes began) and May 2013 (end of study period).

### Information sources

Studies were identified by searching electronic databases, scanning references lists of articles and if relevant findings about factors for non-vaccination of UVGs were found in the full-text articles from the first review, these articles were selected for the second search. However, full-text articles from the first review were only used in the case that no articles about factors for non-vaccination were found for that UVG.

This search was applied to MEDLINE (1950-Present), EMBASE (1950-Present) and PsycINFO (1950-Present). The last search was run on May 2013. For both systematic reviews, we selected - with the assistance of a librarian - a specific search term combination, based on MeSH (Medical Subject Headings) and ‘free text’ (i.e. title and/or abstract) terms.

### Search

The two search strategies are briefly described below. For the first strategy, the search term combination was based on the list of European countries and a list of VPDs and any of the search terms outbreak, epidemic or low vaccination coverage and any of the search terms community, minority, ethnic, group, or subgroup.

For the second strategy, the search term combination included the list of European countries, a list of VPDs and a list of the names (including other terms referring to the same group) of UVGs found in the first literature review and any of the search terms ethnic groups, minority groups, religion, anthroposophic, or complementary therapies and any of the search terms attitude, belief, argument, treatment refusal, patient acceptance of health care, “health knowledge, attitude, practice”, decision making, patient compliance, ideology, or objection. Tables 1 and 2 present the full electronic search strategy for the MEDLINE database.

**Table 1:** Full electronic search strategy for the MEDLINE database – Identification of UVGs

Database: MEDLINE 1950 to present, MEDLINE In-Process & Other Non-Indexed Citations Search Strategy	
1	exp disease outbreaks/sn (5773)
2	exp disease outbreaks/ep (3512)
3	exp disease outbreaks/pc (12003)
4	exp *Disease Outbreaks/ (42825)
5	1 or 2 or 3 or 4 (47887)
6	exp population groups/ (197252)
7	exp population surveillance/ (48732)
8	exp measles/ (12908)
9	exp measles vaccine/ (7121)
10	exp Measles-Mumps-Rubella Vaccine/ (2056)
11	exp mumps/ (3574)
12	exp mumps vaccine/ (2965)
13	exp rubella/ (7276)
14	exp rubella vaccine/ (4256)
15	exp influenza human/ (33423)
16	exp influenza vaccine/ (15305)
17	exp influenza vaccines/ (15305)
18	exp poliomyelitis/ (15592)
19	exp poliovirus vaccines/ (6073)
20	exp whooping cough/ (6302)
21	exp tetanus/ (8118)
22	exp Diphtheria-Tetanus-Pertussis Vaccine/ (2306)
23	exp Diphtheria/ (5187)
24	exp pertussis vaccine/ (6539)
25	exp diphtheria tetanus vaccine/ (307)
26	exp meningococcal infections/ (9327)
27	exp meningococcal vaccines/ (2232)
28	exp hepatitis b/ (43927)
29	exp hepatitis b vaccines/ (7243)
30	exp hepatitis b virus/ (18871)
31	exp pneumococcal infections/ (16122)
32	exp pneumococcal vaccines/ (4460)
33	8 or 11 or 13 or 15 or 18 or 20 or 21 or 23 or 26 or 28 or 30 or 31 (160650)
34	9 or 10 or 12 or 14 or 16 or 17 or 19 or 22 or 24 or 25 or 27 or 29 or 32 (47477)
35	exp vaccines/ (165840)
36	exp vaccination/ (54688)
37	exp mass vaccination/ (1849)
38	33 and (35 or 36 or 37) (38389)
39	5 and (6 or 7) and (34 or 38) (640)
40	39 (640)
41	(vaccin$ adj (rate$ or coverage$)).ti,ab. (5377)
42	(low adj vaccinat$).ti,ab. (211)
43	40 and (41 or 42) (105)
44	exp immunization programs/ (8797)
45	5 and (34 or 38) (4269)
46	(group$ or ethnic$ or minorit$ or communit$ or subgroup$).ti. (277479)
47	45 and 46 (173)
48	47 (173)
49	(ethnic$ or minorit$ or communit$ or subgroup$).ti,ab. (518807)
50	45 and 49 (394)
51	exp disease susceptibility/ (103745)
52	exp health services accessibility/ (79608)
53	exp vulnerable populations/ (5086)
54	exp patient acceptance of healthcare/ (154409)
55	exp treatment refusal/ (10295)
56	exp minority groups/ (9817)
57	exp attitude to health/ (275774)
58	50 and (51 or 52 or 53 or 54 or 55 or 56 or 57) (52)
59	58 (52)
60	45 and 53 (10)
61	45 and 56 (2)
62	60 or 61 (12)
63	(34 or 38) and outbreak$.ti. and ((ethnic$ or minorit$ or communit$ or subgroup$).ti. or exp. *patient acceptance of healthcare/ or exp. *treatment refusal/ or exp. *minority groups/) (65)
64	63 (65)
65	(34 or 38) and outbreak$.ti. and 57 (31)
66	65 (31)
67	exp treatment refusal/ (10295)
68	exp “religion and medicine”/ (9768)
69	(34 or 38) and outbreak$.ti. and 68 (17)
70	(34 or 38) and outbreak$1.ti. and 67 (18)
71	69 or 70 (31)
72	71 (31)
73	(34 or 38) and (exp *treatment refusal/ or exp. *“religion and medicine”/) (116)
74	73 (116)
75	outbreak$.ti,ab. and (72 or 74) (35)
76	(united adj states).ti. (36958)
77	75 not 76 (33)
78	77 (33)
79	43 or 59 or 62 (158)
80	79 and (exp *health services accessibility/ or exp. *minority groups/ or exp. *attitude to health/ or exp. *treatment refusal/ or exp. *“religion and medicine”/) (27)
81	64 or 66 or 72 or 78 (97)
82	81 or 59 or 62 or 80 (143)
83	82 (143)
84	83 not 76 (140)
85	(Peru or Nigeria or Sydney or Brazil or Zealand or Mexico or Australia$).ti. (91932)
86	84 not 85 (127)
87	(35 or 36 or 37) and (exp *health services accessibility/ or exp. *minority groups/ or exp. *attitude to health/ or exp. *treatment refusal/ or exp. *“religion and medicine”/) and outbreak$.ti. (24)
88	87 (24)
89	88 not 86 (4)
90	89 not 76 (2)
91	exp *treatment refusal/ and exp. *“religion and medicine”/ (114)
92	91 and (35 or 36 or 37) (5)
93	92 (5)
94	67 and 68 and (35 or 36 or 37) and outbreak$.ti,ab. (4)
95	94 (4)
96	33 and 68 (76)
97	34 and 68 (26)
98	96 or 97 (82)
99	98 (82)
100	99 not (86 or 90 or 93 or 95) (62)
101	100 not (76 or 85) (57)
102	86 or 90 or 93 or 95 or 101 (189)
103	(measles and outbreaks).ti. (97)
104	103 and italy.ti. (1)
105	(34 or 38) and outbreak$.ti. and coverage$.ti. (22)
106	105 (22)
107	106 not 102 (19)
108	exp ethnic groups/ (105533)
109	4 and exp. *ethnic groups/ and 33 (36)
110	109 (36)
111	110 not 102 (25)
112	(minority and coverage).ti. and (35 or 36 or 37) (2)
113	exp *minority groups/ and (35 or 36 or 37) and coverage.ti. (2)
114	(religious and vaccin$ and coverage).ti. (1)
115	exp *Immunization Programs/ (5694)
116	115 and 68 (18)
117	116 (18)
118	117 not 102 (12)
119	107 or 111 or 113 or 118 (58)
120	119 not (76 or 85) (53)
121	4 and 33 (10049)
122	exp hepatitis a/ (17195)
123	exp dysentery/ (10986)
124	exp shigella/ (10261)
125	33 or 122 or 123 or 124 (191696)
126	4 and 125 (11161)
127	7 and 126 (1495)
128	127 (1495)
129	(56 or 108) and 126 (100)
130	129 (100)
131	128 or 130 (1579)
132	131 (1579)
133	exp anthroposophy/ (180)
134	epidemiology.fs. (1122530)
135	exp *measles/ or exp. *mumps/ or exp. *rubella/ or exp. *influenza human/ or exp. *poliomyelitis/ or exp. *whooping cough/ or exp. *tetanus/ or exp. *Diphtheria/ or exp. *meningococcal infections/ or exp. *hepatitis b/ or exp. *hepatitis b virus/ or exp. *pneumococcal infections/ (131969)
136	exp *hepatitis a/ or exp. *dysentery/ or exp. *shigella/ (27317)
137	135 or 136 (157514)
138	exp europe/ (1059556)
139	132 and (137 or 133) and 138 and 134 (693)
140	139 (693)
141	(outbreak or population or (low adj vaccination)).ti. (153831)
142	140 and 141 (211)
143	140 (693)
144	limit 143 to “review articles” (17)
145	142 or 144 (228)
146	exp Treatment Refusal/ (10295)
147	140 and (133 or 146) (5)
148	132 and (133 or 146) (7)
149	145 or 148 (230)
150	(measles$ or mumps$ or rubella$ or influenza$ or poliomyelitis$ or (whooping adj cough) or tetanus$ or diphtheria$ or pertussis$ or meningococcal$ or hepatitis$ or pneumococcal$ or dysentery$ or shigella$).ti. (245343)
151	(outbreak$ or epidemic$ or denominat$).ti. (45258)
152	(minorit$ or (isolated adj group$) or (low adj vaccinat$) or (vaccine$ adj (rate or rates or coverage$)) or ethnic$ or communit$ or (treatment adj refus$) or religious$ or gipsy or gipsies or anthropo$).ti. (131353)
153	150 and 151 (8602)
154	152 and 153 (276)
155	154 not (102 or 120 or 149) (191)
156	102 or 120 or 149 or 154 (644)
157	remove duplicates from 156 (591)

**Table 2:** Full electronic search strategy for the MEDLINE database – Factors (beliefs, attitudes and reasons) of UVGs regarding vaccination

Database: MEDLINE 1950 to present, MEDLINE In-Process & Other Non-Indexed Citations Search Strategy:	
1	exp measles/ (12908)
2	exp measles vaccine/ (7121)
3	exp Measles-Mumps-Rubella Vaccine/ (2056)
4	exp mumps/ (3574)
5	exp mumps vaccine/ (2965)
6	exp rubella/ (7276)
7	exp rubella vaccine/ (4256)
8	exp influenza human/ (33423)
9	exp influenza vaccine/ (15305)
10	exp influenza vaccines/ (15305)
11	exp poliomyelitis/ (15592)
12	exp poliovirus vaccines/ (6073)
13	exp whooping cough/ (6302)
14	exp tetanus/ (8118)
15	exp Diphtheria-Tetanus-Pertussis Vaccine/ (2306)
16	exp Diphtheria/ (5187)
17	exp pertussis vaccine/ (6539)
18	exp diphtheria tetanus vaccine/ (307)
19	exp meningococcal infections/ (9327)
20	exp meningococcal vaccines/ (2232)
21	exp hepatitis b/ (43927)
22	exp hepatitis b vaccines/ (7243)
23	exp hepatitis b virus/ (18871)
24	exp pneumococcal infections/ (16122)
25	exp pneumococcal vaccines/ (4460)
26	1 or 4 or 6 or 8 or 11 or 13 or 14 or 16 or 19 or 21 or 23 or 24 (160650)
27	2 or 3 or 5 or 7 or 9 or 10 or 12 or 15 or 17 or 18 or 20 or 22 or 25 (47477)
28	exp vaccines/ (165840)
29	exp vaccination/ (54688)
30	exp mass vaccination/ (1849)
31	exp hepatitis a/ (17195)
32	exp dysentery/ (10986)
33	exp shigella/ (10261)
34	(26 or 31 or 32 or 33) and (28 or 29 or 30) (40134)
35	27 or 34 (58398)
36	exp Treatment Refusal/ (10295)
37	exp Health Knowledge, Attitudes, Practice/ (66897)
38	exp “Patient Acceptance of Health Care”/ (154409)
39	exp Attitude to Health/ (275774)
40	exp “Attitude of Health Personnel”/ (114245)
41	(argument$ or belief$ or ideolog$ or attitud$ or objection$).ti. (44532)
42	exp Ethnic Groups/ (105533)
43	exp Minority Groups/ (9817)
44	exp Religion/ (45812)
45	exp Immunization Programs/ (8797)
46	exp Complementary Therapies/ (168626)
47	exp Vaccination/ (54688)
48	exp Decision Making/ (112804)
49	exp Patient Compliance/ (50195)
50	exp Immunization/ (131148)
51	anthroposoph$.ti. (130)
52	35 or 45 or 47 or 50 (165486)
53	36 or 37 or 38 or 39 or 40 or 41 or 48 or 49 (478347)
54	42 or 43 or 44 or 46 or 51 or homeopath$.ti. (316493)
55	52 and 53 and 54 (324)
56	exp africa/ or exp. americas/ or exp. asia/ or exp. australia/ (1996966)
57	(vaccin$ or immuniz$ or immunis$ or measle$ or mumps or rubella$ or polio or mmr).ti. (157864)
58	(relig$ or orthodox$ or protestant$ or racial$ or anthropos$ or refusal or gypsy or gypsies or homeopat$ or homoeopat$ or jewish$ or minorit$ or multiethnic$ or unorthodox$ or latino or ideolog$ or ethnicity or (ethnic adj background) or (alternative adj medicine$) or (parental adj refusal$)).ti. (37918)
59	57 and 58 (310)
60	(55 or 59) not 56 (202)
61	remove duplicates from 60 (193)
	NEW (set 62 is the “new” set 58):
62	(relig$ or orthodox$ or protestant$ or racial$ or anthropos$ or refusal or gypsy or gypsies or homeopat$ or homoeopat$ or jewish$ or minorit$ or multiethnic$ or unorthodox$ or latino or roma or sinti$ or traveler$ or tinker$ or ideolog$ or ethnicity or (ethnic adj background) or (alternative adj medicine$) or (parental adj refusal$)).ti. (40501)
63	57 and 62 (501)
64	(55 or 63) not 56 (346)
65	remove duplicates from 64 (334)
	Search strategy with renewed set 58
1	exp measles/ (12908)
2	exp measles vaccine/ (7121)
3	exp Measles-Mumps-Rubella Vaccine/ (2056)
4	exp mumps/ (3574)
5	exp mumps vaccine/ (2965)
6	exp rubella/ (7276)
7	exp rubella vaccine/ (4256)
8	exp influenza human/ (33423)
9	exp influenza vaccine/ (15305)
10	exp influenza vaccines/ (15305)
11	exp poliomyelitis/ (15592)
12	exp poliovirus vaccines/ (6073)
13	exp whooping cough/ (6302)
14	exp tetanus/ (8118)
15	exp Diphtheria-Tetanus-Pertussis Vaccine/ (2306)
16	exp Diphtheria/ (5187)
17	exp pertussis vaccine/ (6539)
18	exp diphtheria tetanus vaccine/ (307)
19	exp meningococcal infections/ (9327)
20	exp meningococcal vaccines/ (2232)
21	exp hepatitis b/ (43927)
22	exp hepatitis b vaccines/ (7243)
23	exp hepatitis b virus/ (18871)
24	exp pneumococcal infections/ (16122)
25	exp pneumococcal vaccines/ (4460)
26	1 or 4 or 6 or 8 or 11 or 13 or 14 or 16 or 19 or 21 or 23 or 24 (160650)
27	2 or 3 or 5 or 7 or 9 or 10 or 12 or 15 or 17 or 18 or 20 or 22 or 25 (47477)
28	exp vaccines/ (165840)
29	exp vaccination/ (54688)
30	exp mass vaccination/ (1849)
31	exp hepatitis a/ (17195)
32	exp dysentery/ (10986)
33	exp shigella/ (10261)
34	(26 or 31 or 32 or 33) and (28 or 29 or 30) (40134)
35	27 or 34 (58398)
36	exp Treatment Refusal/ (10295)
37	exp Health Knowledge, Attitudes, Practice/ (66897)
38	exp “Patient Acceptance of Health Care”/ (154409)
39	exp Attitude to Health/ (275774)
40	exp “Attitude of Health Personnel”/ (114245)
41	(argument$ or belief$ or ideolog$ or attitud$ or objection$).ti. (44532)
42	exp Ethnic Groups/ (105533)
43	exp Minority Groups/ (9817)
44	exp Religion/ (45812)
45	exp Immunization Programs/ (8797)
46	exp Complementary Therapies/ (168626)
47	exp Vaccination/ (54688)
48	exp Decision Making/ (112804)
49	exp Patient Compliance/ (50195)
50	exp Immunization/ (131148)
51	anthroposoph$.ti. (130)
52	35 or 45 or 47 or 50 (165486)
53	36 or 37 or 38 or 39 or 40 or 41 or 48 or 49 (478347)
54	42 or 43 or 44 or 46 or 51 or homeopath$.ti. (316493)
55	52 and 53 and 54 (324)
56	exp africa/ or exp. americas/ or exp. asia/ or exp. australia/ (1996966)
57	(vaccin$ or immuniz$ or immunis$ or measle$ or mumps or rubella$ or polio or mmr).ti. (157864)
58	(relig$ or orthodox$ or protestant$ or racial$ or anthropos$ or refusal or gypsy or gypsies or homeopat$ or homoeopat$ or jewish$ or minorit$ or multiethnic$ or unorthodox$ or latino or roma or sinti$ or traveler$ or tinker$ or ideolog$ or ethnicity or (ethnic adj background) or (alternative adj medicine$) or (parental adj refusal$)).ti. (40501)
59	57 and 58 (501)
60	(55 or 59) not 56 (346)
61	remove duplicates from 60 (334)

### Study selection

Two reviewers (NF and LM) independently selected the relevant articles according to the case definition of a UVG and search terms. Firstly, the selection was based on title and abstract for papers identified in MEDLINE and only on title for papers identified in the two other databases. The final selection was done on full-paper. In case of discrepancy, a third reviewer (JS) was asked to review articles. All the reviewers discussed the findings and consensus was reached.

### Data collection process

A data extraction sheet was developed by the first author where title manuscript, publication year, name of UVG, short description of UVG (i.e. population size and main characteristics), which country or countries in Europe they live, which VPDs have been circulating, based on what it was clear that it was a UVG (outbreak/seroprevalence/vaccination coverage), vaccination coverage, which beliefs, attitudes and reasons for non-vaccination were described. The form was not piloted and extractions were not completed in duplicate.

### Syntheses of results

For the first review we provided a short description of each UVG such as the population size and in which countries members of the UVG live and their main characteristics. In addition, other information such as about outbreaks that have occurred in that population and estimations of the vaccination coverage were provided. For the second review we first described beliefs, attitudes and reasons for non-vaccination among each UVG found in the literature. Secondly, we counted the number of times a certain factor was mentioned in the articles found for each UVG. Note, if a factor was reported in two articles it does not have to mean that the factor was twice more important than when a factor was reported in only one article. If it was a qualitative study we took each factor mentioned in that study once into account. If it was a quantitative study we took the factor into account if equal or more than 80% of the study population agreed with that factor. If different articles described the same population then similar factors were only taken into account once.

## Results

### Identification of the under-vaccinated groups in Europe

**Fig. 1:**
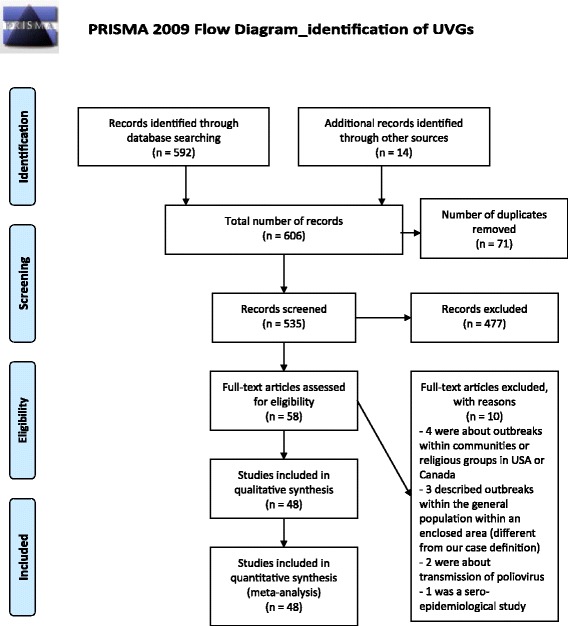
Prisma flow chart for search and selection of articles – Identification of UVGs

The first literature search resulted in 606 articles (Fig. 1). These were screened based on abstract and/or title, and 58 of them were subsequently screened based on full-text article. Of them, 48 were selected and included in the review (Fig. 1). Thirteen articles were found in the index references of 34 selected articles, and 1 came from the second literature search. Consequently, 48 articles were selected.

We identified five UVGs: Orthodox Protestants (11 articles), Anthroposophists (9 articles), Roma (18 articles), Irish Travellers (7 articles), and Orthodox Jewish communities (8 articles) (2 articles mentioned 2 UVGs and 1 article mentioned 4 UVGs, thus totalling 48). Below we briefly describe the five UVGs.

Practically all articles (47 of 48) were published after 1990. Four describe vaccination coverage among Orthodox Protestants, Irish Travellers or Orthodox Jewish children. The remaining articles (*n* = 44) were outbreak reports describing mainly outbreaks occurring between 2004 and 2012, including 35 articles describing measles outbreaks.

### Orthodox Protestant communities

Orthodox Protestants (OP) live in close-knit communities within Dutch society with their own church, political party, primary and secondary schools. The population size is estimated at 250,000, i.e. 1.5% of the Dutch population [13, 14] and almost 75% of them live in an area stretching from the south-west to the north-east of the Netherlands, also called ‘Bible belt’. Different OP denominations vary in their interpretation of the Bible.

The overall vaccination coverage among OPs is estimated to be at least 60% but it varies from below 25% to more than 85%, depending on the OP denomination [13]. In another study identified by the literature review, comparing the percentage of 2 year-olds who completed Diphtheria Tetanus Pertussis Polio vaccination, the mean vaccination coverage was estimated to be 93.5% (± 4.7) in municipalities with one or more OP denominations, which was significantly lower than in municipalities without OP denominations (96.9% ± 2.1) [14].

In the Netherlands, from 1950 until May 2013, different outbreaks are described in the literature within this community such as poliomyelitis in 1978 [15] and 1992–1993 [16], and mumps [17, 18], measles [8, 9], and rubella [3, 5, 6] outbreaks between 1999 and 2009.

### Anthroposophists

Anthroposophy is a spiritual movement founded at the beginning of the twentieth century by Rudolf Steiner (1861–1925), an Austrian philosopher, social reformer, architect and esotericist. Anthroposophists applied his theory to different settings such as education, medicine, architecture and agriculture. The Anthroposophical Society has its international centre at the Goetheanum in Switzerland. They have developed schools (222 in Germany and 464 in other European countries), anthroposophical health care centres and centres for people with learning disabilities [2, 19]. Anthroposophists live in various countries over the world and in almost all European countries. The number of individuals sharing anthroposophical beliefs is unknown. No articles were identified about the vaccination coverage among this group. They experienced outbreaks of measles in United Kingdom in 1997 [19], in Austria [20–22], Germany [7, 22, 23], Norway [22] and measles and mumps outbreaks in the Netherlands [18, 24] between 2008 and 2010.

### Roma

Roma constitute a transnational ethnic community composed of various groups (e.g. Kalderash, Lovari, Churari, Romanichal) living predominantly in Central and South-Eastern Europe [2]. The size of the Roma population within the European Union is estimated to be in the range of 6–8 million people [2, 25]. The actual figure might be even higher given that there is no agreed upon definition of who is Roma [25], a part of the community is highly mobile, and some people who self-identify as Roma are reluctant to disclose their ethnicity during census for fear of stigmatization [2]. Roma have been historically marginalized [2] and still face significant discrimination nowadays [25, 26]. Their health indicators are significantly worse than those of the general population [2]. The poor economic conditions and improper housing [27] create favourable circumstances for the spread of communicable diseases. The Roma community usually has low vaccination coverage [25]. Since 2006, several measles outbreaks occurred within their communities in Italy [28, 29], Germany [30–32], Greece [33, 34], Romania [35], Croatia [36], Serbia [37], Poland [26], Bulgaria [27], Ireland [38] and Spain [39–41].

### Irish Travellers

Irish Travellers are also called Travelling community or Gypsy-Travellers and are recognised as an ethnic minority group in the United Kingdom (UK) and Ireland; all of whom were, or are, nomadic [2, 42]. Today, although nomadism is an important part of their culture and history, the term is more accurately a descriptor of ethnic identity, distinct beliefs and culture (language, traditions, social organisation), rather than a description of actual daily activities [2, 42]. Their number is difficult to estimate and reports vary widely (in 2008, from 82,000 to 300,000 in England & Wales, and around 40,129 in Ireland) [2]. Many are reluctant to disclose their ethnic identity due to fears of prejudice and mistrust of authority. They often have poor access to education and employment. In the sphere of/regarding health care, the marginalisation and their travelling way of life has historically resulted in poor access to services – including immunisation [2]. The Irish Traveller community usually has low vaccination coverage [2, 42–46]. Outbreaks of measles in Irish Traveller communities are well recognised in the European region, particularly in the UK. A measles outbreak associated with a gathering of Irish Travellers in 2007 in England was subsequently linked to a measles outbreak in Norway among nomadic Irish Travellers from England [42–45]. Another measles outbreak occurred in Ireland in 2009 among this community, which was also reported among the Roma community and spread to the general population in 2010 [38].

### Orthodox Jewish communities

The Orthodox Jewish (OJ) community shares religious observance and cultural practices. They are usually living closely within their own community, have large families with a high proportion of young children and often have considerable household crowding [2, 47]. In Europe, there are significant OJ communities in London (the largest community, with over 20,000 members) and Salford in the UK, and in Antwerp in Belgium (10,000 members) [47, 48].

No articles were identified about the overall vaccination coverage among this group. In a 1991–1992 study in north east London [49], the vaccination coverage was 79% for Measles Mumps Rubella (MMR) (95%CI: 75–85) among OJ children, similar to the coverage in the general population. Two measles outbreaks happened within this community in 2007–2008 in Belgium [50, 51] and England [51, 52]. These outbreaks were epidemiologically linked, and spread to Israel [53]. Many of these children were incompletely immunised [50].

### Description of ideologies, beliefs or attitudes towards vaccination among the identified UVGs

**Fig. 2:**
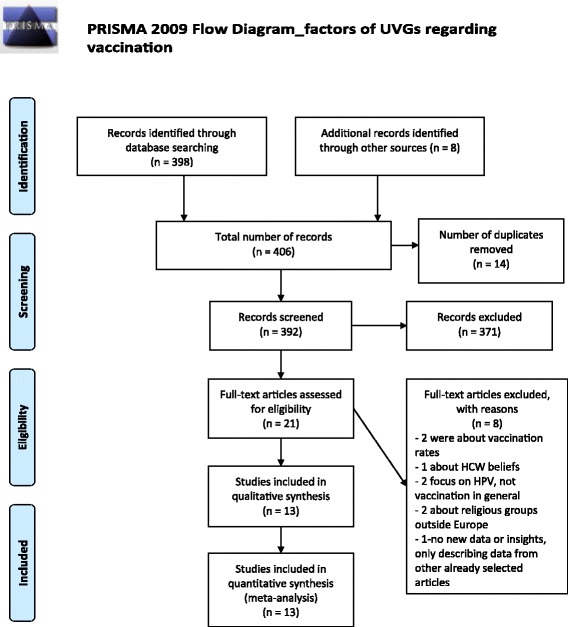
Prisma flow chart for search and selection of articles – Factors (beliefs, attitudes and reasons) of UVGs regarding vaccination

The second literature search resulted in 406 articles (Fig. 2). This resulted in 21 articles that were screened based on full-text article, and 13 were subsequently selected and included in the review (Fig. 2): one about Orthodox Protestants, four about Roma, three about Irish Travellers and 1 about both groups, two about Anthroposophists, and two about Orthodox Jewish communities. Despite that no English language articles were found specifically addressing beliefs, attitudes and reasons towards vaccination as a main topic among Irish Travellers and Roma communities, eight articles were found in the first literature search, which also briefly described factors for non-vaccination that lead to the corresponding outbreak in these two communities. In total, 13 articles were included in the second literature search. The list of factors regarding vaccination (beliefs, attitudes and reasons) for each UVG is presented in Table 3 and below the factors are briefly explained per UVG.

**Table 3:** List of reported factors (beliefs, attitudes and reasons) regarding vaccination and vaccine preventable diseases (VPD) among the five identified UVGs. The numbers stand for the number of times a certain factor was mentioned in the articles found for each UVG

	Orthodox Protestants (1 study)	Anthroposophists (2 studies)	Roma (5 studies)	Irish Travelers (4 studies)	Orthodox Jewish (2 studies)
*Perceived severity/susceptibility of VPD*					
Perceived non-severity of VPD:					
- some VPD are not severe (e.g. not severe: measles, mumps, pertussis; severe: tetanus, polio and diphtheria)	1	1		1	
- some VPD are helpful for child’s development (e.g. measles)		2		1	
Perceived non-susceptibility to VPD (e.g. only a small number of children with VPD disease)		1			
*Perceived safety/effectiveness of vaccine*					
Perceived un-safety of the vaccine (adverse events, misconceptions)	1	1		2	1
Perceived non-effectiveness of the vaccine (e.g. graphs and reports do not prove effectiveness of the vaccines)		2			
Beliefs about vaccine (components of the vaccine could be dangerous e.g. poisons, toxins, contaminants)		1			1
*Flexibility / freedom*					
Adapting vaccination schedule to the child (schedule not flexible enough)		1			
Combined vaccines should be monovalent (parents like to choose, parents perceive overload of antigens of various diseases in combination vaccine)		1			
*Knowledge*					
Need for more information or lack of information (e.g. risk of vaccination / scientific facts from the Public Health authorities, where and when to vaccinate)		1	1	2	1
Low awareness of vaccination as a preventive measure			1		
*Social networks* *(parents heard term MMR linked with fears)*					1
*Ideas about prevention*					
Anthroposophic (healthy lifestyle, confidence in the health of the child, freedom of choice in healthcare and natural remedies, let the body experience certain infections)		2			
Complementary medicine being unavailable					1
Religious beliefs (trust in God)	1				1
*Social structural aspects*					
Socio-economic and cultural differences (e.g. language barriers, improper housing, low level of formal education, illiteracy)			4	3	
Improvement in access and facilities for their children (e.g. limited and inflexible clinic hours)					1
Poor access to health care centres (e.g. high spatial mobility for Roma and Irish Travellers)			4	3	
Exposure to stigmatization, marginalization and discrimination			3	3	
No trust in information from the Public Health authorities		1		1	

### Orthodox Protestant communities

One Dutch study among Orthodox Protestant (OP) parents [54] used in-depth and semi-structured interviews with 27 families. The aim of this study was to gain insight into how OP parents decide for or against vaccinating their children. Four different groups emerged from this study: traditionally non-vaccinating, deliberately non-vaccinating, traditionally vaccinating, and deliberately vaccinating parents. The main argument for those who refuse vaccination was the necessity to rely on Divine providence: if God sends an illness to somebody or an outbreak on earth, he has a reason to do so. One must not oppose God’s will and should trust in God. On the contrary, those who actively choose to vaccinate their child(ren) consider that vaccination is a gift from God. Some members were concerned about vaccine safety (i.e. about the disease inducing properties of vaccines) and side-effects and some did not consider some paediatric illnesses as serious diseases such as measles and mumps.

### Anthroposophists

Two studies are related to Anthroposophists. One study [55] was performed in the Netherlands and used focus-group methodology among parents who visited an anthroposophical Child Welfare Centre. Another study [56] was performed in the UK and sent postal questionnaires to measles cases from a predominantly un-immunised anthroposophical community in Gloucestershire.

Parents with an anthroposophical view believed that with a healthy life, a good nutrition (e.g., breastfeeding for babies), and a safe environment (e.g. mothers who stay at home to take care of their children) the immune system of children would be stronger and better able to fight against infectious diseases and therefore vaccination is not needed. Some carefully weighed the perceived severity of and susceptibility to the infection in making their vaccination decision [55]. Some parents believed that paediatric illnesses are necessary and a part of the development of the child. Some parents had doubts about the safety, the side-effects, the effectiveness and the components of the vaccine [55, 56]. Most of the parents preferred monovalent (single antigen) vaccines instead of combination vaccines in order to have more freedom of choice and flexibility to adapt the schedule. In the Dutch study, while they mostly trusted health care providers, some did not trust the information provided by the Public Health authorities. All parents mentioned the need to have more information about the risk of vaccinating, the components of the vaccine and more transparency from the Public Health authorities [55].

### Roma

No article in the sample elaborates on Roma attitudes and beliefs regarding vaccination. There is no evidence that Roma parents object on ideological grounds to having their children immunized. Their misconceptions about vaccination are by no means different from those encountered in the general population (e.g. lack of information and misconceptions about vaccine safety) [2]. For a variety of reasons, many members of the community have difficulties in accessing healthcare services [2, 25]. The explanations advanced by the authors for the low vaccination uptake among Roma include the high spatial mobility of some members of this ethnic community [25, 27], which makes them difficult to reach by vaccination programmes; precarious socio-economic conditions [2, 26, 29] and exposure to stigmatization, marginalization, and discrimination [2, 25, 26]; low level of formal education [26]; low awareness of vaccination as a preventive measure [26]; and cultural differences from the general population (e.g. language barriers, religious beliefs, traditional remedies, practice of early marriages, lower social position of women in Roma communities) [26].

### Irish Travellers

From the second literature review, also no English language article was found referring to attitudes, beliefs and reasons regarding vaccination within the Irish Travellers community. However, some reasons for low vaccination coverage were reported during measles outbreaks in several countries in Europe [2, 42]. In the 2007 measles outbreak among Irish Travellers in London with links to a Norwegian measles outbreak, the Norwegian authorities reported that the Irish Traveller community responded favourably to interventions, with many non-vaccinated contacts being given MMR vaccine [44], suggesting that the community is not averse to vaccination in general. Similar misconceptions about vaccination are present among Irish Travellers compared to the general population (e.g. lack of information and misconceptions about vaccine safety) [2]. Additionally, Muscat et al. [2] suggested that the low vaccination coverage among this community is explained by poor access to health care because of population mobility. Other identified barriers to healthcare access – including access to immunisation services - for Irish Travellers include inequalities in registration with family doctors (due to discrimination, mismatch in expectations, confusion about requirements for registration), illiteracy, and lack of services that are culturally sensitive and respond to the needs of Traveller communities [2, 42, 46].

### Orthodox Jewish communities

Two studies, using semi-structured interviews or administered questionnaires, were conducted in north-east London among Orthodox Jewish families. Both aimed to identify reasons for low uptake of immunisation [50, 57]. In the study from Cuninghame in 1991–1992 [49], parents deemed immunisation important and that measles could be a serious illness. The main reason for missing immunisation was parental decisions to delay immunisation, usually MMR. Some had concerns about side-effects of the vaccine. Contrary to these findings, in the study from Henderson, conducted in 2003 [57], some mothers believed that BCG vaccination was unnecessary because they were living separately from the general population and consequently were not exposed. Some others had doubts about the Measles Mumps Rubella (MMR) or Diphtheria Polio Tetanus (DTP) vaccination. Despite their lack of exposure to a broader social network, ideas spread by media reached them and therefore, they felt anxiety about vaccination. They were afraid of adverse effects from MMR and whooping cough vaccinations and to have foreign substances associated with illness being injected into their child. Religious arguments were also reported. They trust in God and if God wants to give a disease, the child will get it. Both articles identified barriers to accessing health care/ vaccinations including: restricted practice opening time, lengthy waiting times and the difficulty to rearrange appointments.

## Discussion

Public Health authorities in the European region face challenges with outbreaks among UVGs, and equally in efforts to meet the requirements set by the WHO for eliminating measles and rubella in the near future. Elimination can only be obtained through high vaccination coverage in the respective countries. As long as pockets of low vaccination coverage remain in many European countries, outbreaks of VPDs will continue to occur, and elimination will be infeasible as long as there are UVGs. These pockets of low vaccination coverage may occur through clustering once exemption begins to take hold in a particular community [58]. Besides outbreaks will occur within these groups, they can also act as a source for further transmission to the general population. Increasing vaccination uptake in these groups starts with talking with the groups and develop together with them appropriate communication. We found several common beliefs related to non-vaccination in these groups that could help to find policy vantage points for communication with these groups.

Looking at outbreaks and low vaccination coverage studies among groups and communities, we identified five UVGs throughout Europe that represent a significant number of people: Orthodox Protestants in the Netherlands [13, 14] (around 250,000 persons), Anthroposophists mostly in Austria, Germany and bordering countries (numbers not available), Roma mainly in Central and South-eastern Europe [2, 25] (6–8 million), Irish Travellers in the United Kingdom and Republic of Ireland [2] (120,000–340,000) and Jewish Orthodox in the United Kingdom and Belgium [47, 48] (around 30,000). All five UVGs experienced measles outbreaks. Measles is highly contagious and requires at least 95% vaccination coverage to maintain herd immunity [59], which could explain the higher number of measles outbreaks (35) that were described compared to rubella (3) and mumps (2) outbreaks. In the context of low vaccination coverage, large VPD outbreaks will continue to occur in these groups, with the risk of spreading disease to vulnerable individuals in the general population (vaccinated or not), as has been seen in several previous measles outbreaks [8].

From the factors-oriented literature search, we conclude that there is as yet little published English-language literature specifically addressing beliefs, attitudes and reasons regarding non-vaccination among these groups, as we only found five articles. These five articles were based on empirical research, using both qualitative and quantitative study designs: one among Orthodox Protestant parents, two among Anthroposophists and two among Orthodox Jewish parents. We have not identified research published in the English international literature aiming to study mainly beliefs, attitudes and reasons regarding non-vaccination among the Roma and the Irish Travellers communities. However, some reasons for non-vaccination were described in the eight additional articles selected from the first literature search. A variety of beliefs and objections to vaccination were reported among each group. Not all members have the same beliefs, also called within-group heterogeneity. On the other hand, some similar beliefs were shared between different groups, also called between-group homogeneity. The most frequently mentioned shared reasons for not vaccinating their children are: the perceived non-severity of the disease, the perceived un-safety of the vaccine (e.g. the fear of side effects and misconceptions), and the need for more information or the lack of information about for example risks of vaccination. Apart from these common factors for non-vaccination, each UVG has its own specific factors. Low vaccination coverage for certain diseases among the Anthroposophists could be explained by their specific philosophy of a healthy lifestyle. For Orthodox Protestant, firm trust in Divine Providence seems to be the most important reason for not being vaccinated. This religious factor is incidentally also found among the Orthodox Jewish communities, although Jewish scholars have rejected arguments that medical interventions interfere with divine providence [60]. Whereas low vaccination coverage among Anthroposophist, Orthodox Protestants and Orthodox Jewish communities may be explained by their beliefs, our findings from the literature suggest that low vaccination coverage among Roma and the Irish Travellers communities is predominantly explained by poor access to health care services due to mobility.

As the objective of our contribution to the EU project mentioned in the introduction was to find vantage points for communication tactics with UVGs in case of an epidemic, we searched for factors (beliefs and attitudes) regarding non-vaccination of these groups with regard to epidemics. No literature has been found on UVGs in the framework of pandemics, also not with regard to the influenza A(H1N1) pandemic in 2009. We did find literature on factors for non-vaccination against pandemic influenza A among the general population as the general vaccination uptake during the 2009 pandemic was low in various countries [61]. Strikingly, most of the factors for non-acceptance among UVGs of regular universal childhood vaccines (e.g. un-safety/fear of adverse events, non-severity of the disease, lack of information about risk of vaccination) were similar to those among the general population in various countries during the A(H1N1) pandemic [62, 63] as well as for routine universal vaccinations [46, 64–72]. The same factors were also found on many anti-vaccination websites [73–76] opposing routine universal vaccination. New forms of reluctance to vaccinate seem to emerge in the general population, identified as people following an alternative dietary system (macrobiotic) and among so called critical citizens [77, 78]. Another phenomenon are the free-riders: in a highly vaccinated population one can avoid vaccine adverse events by non-vaccination while being protected by the vaccinated contacts (herd immunity) [79]. If free-riding takes hold in a social network new under-vaccinated pockets may arise. As these new opponents are (as yet) not organised as a group as defined in this article, we did not include them in the literature review. However, we are aware that these “like-minded groups that are not geographically clustered” might gain importance in the near future as the number of followers seems to be growing. It is therefore important to start engaging with these “like-minded groups that are not geographically clustered” as soon as possible by listening to their arguments and try to mitigate vaccine refusals.

In our opinion, two shared beliefs for non-vaccination, also found in the general population, are amenable for influencing vaccination decisions by targeted communication tactics that are discussed later on: perceived non-severity of the disease and its possible complications, and vaccine un-safety. The factors for non-vaccination for Irish Travellers and Roma communities are different and seem to be related mostly to access to health care and therefore also other interventions than communication are needed.

An important limitation of these two literature reviews is that we limited the search to English language peer-reviewed literature. A lot of knowledge is actually available in the grey literature and in the countries where the UVGs live, as it is the case in the Netherlands for Orthodox Protestants, and in Portugal for Roma [80]. However, this information is not available in the open English language scientific literature. Another limitation is that we restricted the search to vaccination and immunisation in MeSH and title and/or abstract. Vaccination is part of the process of health in general and beliefs and attitudes are closely linked. Therefore, we did not include articles explaining beliefs and attitudes regarding vaccination in the broad framework of health. For example, health-related beliefs for Roma and Irish Travelers communities are well described in many articles [81–84] but the relation to vaccination seems to be less important, as access to health care is the dominant factor in these groups.

Previous work in the UK suggests that indiscriminate population-based interventions, that aim to improve overall uptake of vaccination, are unlikely to reduce social-based inequalities in uptake [85]. There needs to be recognition of the differences between population groups, that different approaches are essential to meet the needs of these groups, and that a specific effort has to be made to reach groups with barriers to vaccination in routine vaccination programmes [85, 86]. We have found that important (changeable) beliefs for non-vaccination are shared by many groups, as well as by the general population. We therefore argue that in developing communication strategies for specific UVGs and the general public, partly the same arguments may be used. However, to reach UVGs it is important to co-operate with these groups and to adapt the information to their specific needs. If used in a trustworthy and reliable context, UVGs can use the information, also from other members in their group who do vaccinate, for deciding on vaccination. Several technical reports and tools were developed by ECDC and WHO for health care professionals and Public Health Institutes to increase vaccination uptake and suggest communications activities such as educating people about risks of vaccinating and not vaccinating, addressing misconceptions, promote positive health outcomes, and partnering with health care workers as they are believed to be a trusted source. These tools target especially MMR [87, 88] and/or the general population [89]. A study by Horne et al., 2015 [90] with commentaries by Betsch et al. 2015 [91] and Horne et al. 2015 [92] have shown that highlighting objective information about the consequences of not vaccinating and countering vaccination myths can positively impact the intention to vaccination of people who are doubting. However, on the other hand Nyhan et al., 2014 [93] showed that attempts to increase concerns about communicable diseases (e.g. fear appeals and narratives) or correct false claims about vaccines may be in some cases counterproductive. It is therefore important to carefully test vaccination messages in a specific group.

Governments and public health authorities might have to take a different stand regarding UVGs: firstly, they could act as sentinel population for early detection of transmission of VPD as the large number of susceptibles increases the chance of disease detection if transmission occurs, secondly, they might also be used as well as sentinel for beliefs/attitudes and reasons for non-vaccination in the general population as some of them might take over similar ideas about vaccination. Therefore, efforts to communicate with these groups should start as soon as possible in all EU-countries. The epitheton “hard-to-reach” should be abandoned, as not all groups are hard-to-reach and each group has its specific reasons and even individuals with in a group may differ for which specific approaches are needed and not general ones. Better that each country determines its own UVGs with their own beliefs and starts to develop trustful relationships.

## Conclusions

Within each UVG identified, there are a variety of health beliefs and objections to vaccination. In addition, similar factors are shared by several of these groups. Communication strategies regarding these similar factors such as educating people about the risks associated with being vaccinated versus not being vaccinated, addressing their concerns, and countering vaccination myths present among members of a specific UVG through a trusted source, can establish a reliable relationship with these groups and increase their vaccination uptake. Furthermore, other interventions such as improving access to health care could certainly increase vaccination uptake in Roma and Irish travellers.

## Abbreviations

MeSH: Medical subject headings
OJ: Orthodox Jewish
OP: Orthodox protestants
SAGE: Strategic advisory group of experts
UVG: Under-vaccinated groups
VPD: Vaccine preventable diseases
WHO: World Health Organisation
WP: Work package
